# Nicotine Exposure during Rodent Pregnancy Alters the Composition of Maternal Gut Microbiota and Abundance of Maternal and Amniotic Short Chain Fatty Acids

**DOI:** 10.3390/metabo12080735

**Published:** 2022-08-09

**Authors:** Jasenka Zubcevic, Jacqueline Watkins, Cindy Lin, Byrell Bautista, Heather M. Hatch, Sergei G. Tevosian, Linda F. Hayward

**Affiliations:** 1 Department of Physiology and Pharmacology, University of Toledo College of Medicine, Toledo, OH 43614, USA; 2 Department of Physiological Sciences, University of Florida College of Veterinary Medicine, Gainesville, FL 32610, USA

**Keywords:** smoking, pregnancy, microbiota, SCFA

## Abstract

Tobacco smoking is the leading cause of preventable death. Numerous reports link smoking in pregnancy with serious adverse outcomes, such as miscarriage, stillbirth, prematurity, low birth weight, perinatal morbidity, and infant mortality. Corollaries of consuming nicotine in pregnancy, separate from smoking, are less explored, and the mechanisms of nicotine action on maternal–fetal communication are poorly understood. This study examined alterations in the maternal gut microbiome in response to nicotine exposure during pregnancy. We report that changes in the maternal gut microbiota milieu are an important intermediary that may mediate the prenatal nicotine exposure effects, affect gene expression, and alter fetal exposure to circulating short-chain fatty acids (SCFAs) and leptin during in utero development.

## 1. Introduction

Smoking during pregnancy is one of the major risk factors for spontaneous miscarriage, premature birth and low infant birth weight ([1,2,3,4]; reviewed in [5]). Following birth, offspring of smokers are more likely to die of sudden infant death syndrome [6,7,8] and have compromised respiratory function [9,10], as well as being at increased risk for obesity [11,12], cardiovascular disease [12,13] and nicotine addiction [14] later in life. Emerging evidence suggests that obesity and cardiovascular disease are linked to changes in the balance of microbes in the gut, as well as the interconnection between the metabolic by-products of these microbes and brain function, or what is often referred to as the gut microbiome–brain axis [15]. The composition of the gut microbiome is established early in life, and includes the potential bacterial exposure in utero [16] and the subsequent transfer of maternal bacteria to the infant during birth and/or lactation [17]. Thus, the predisposition for the offspring of smokers to develop obesity and cardiovascular disease, in addition to addiction-related disorders, may be linked to smoking-related shifts in the maternal microbiome. Indeed, evidence from animal models demonstrates that manipulation of the maternal microbiome in the prenatal period has a significant impact on postnatal disease development [18,19].

At present, little is known about the impact of nicotine or smoking on the gut microbiome in general or, specifically, the impact of nicotine exposure during pregnancy on the maternal microbiome and how this may affect the offspring. Of the few studies that have evaluated the effect of smoking on the gut microbiome in non-pregnant adults, the effects appear to be relatively modest [20,21,22]. The gut microbiome, however, changes significantly during pregnancy [23] and the impact of drug exposure on the gut microbiota is markedly different during the pregnant state versus the non-pregnant state [24]. Since ~15% of women continue to smoke during pregnancy, gaining a better understanding of how nicotine exposure impacts the gut microbiome specifically during pregnancy is critical to finding therapeutic options for offspring born to smokers. 

This study was undertaken to explore the poorly understood changes in the maternal gut microbiome in response to nicotine exposure during pregnancy. Using a well-established rodent model of prenatal nicotine exposure (PNE), we hypothesized that PNE will induce changes in the maternal gut microbiome, which will alter fetal exposure to circulating short chain fatty acids (SCFAs) and leptin during in utero development. SCFAs have multiple functions in cardiovascular health [25,26], including their reported role in modulation of leptin release from adipose tissue cells via activation of select receptors [27]. Thus, maternal gut dysbiosis and sustained changes in SCFA levels may affect the developmental circuits that regulate cardiometabolic homeostasis [28,29,30].

## 2. Methods

### Animals

To test our hypothesis, we used a well-characterized model of PNE in rats [31,32,33]. All animal procedures were approved by the University of Florida IACUC prior to the start of the study (#20180188). Specific pathogen-free male and female Sprague Dawley rats were purchased from Envigo and maintained (12:12 h light: dark cycle) in UF’s animal care facility with food and water ad libitum. Females were purchased at 8 weeks of age and were allowed 1 week to acclimate. At 9 weeks of age, females were randomly assigned to one of 4 treatment groups. Two groups of females were mated, and two groups of females remained virgins. Mating included placing a single female overnight with a male and examined on the following morning for the presence of sperm via vaginal swabs. If sperm was present, the dam was weighed and placed in a separate cage and this period was marked as gestational day (GD) 0. If no sperm was detected, the pair remained together, and the female was examined daily until pregnancy and GD0 were confirmed. 

## 3. Exposure to Nicotine

On GD6 (or an equivalent day in virgin females, ~9 weeks of age), female rats underwent a brief surgery for the placement of a subcutaneous osmotic mini pump while anesthetized to a surgical plane with isoflurane (2.5% in 100% oxygen). Using a sterile technique, a 28-day osmotic mini-pump (2ML4-ALZET, Durect Corp., Cupertino, CA, USA) filled with either sterile saline (control; CON) or a nicotine tartrate (6 mg/kg; NIC), dissolved in sterile saline [34,35,36], was placed subcutaneously between the scapulae, as described before [37,38]. In rats, embryo implantation in the uterine wall begins on day 5 and is complete by day 7 within a normal 22-day gestation cycle [39]. The chosen dose of NIC has previously been reported to induce maternal plasma NIC levels associated with moderate smoking [39,40,41] and is the sub-threshold for the induction of overt changes in litter size [35] or fetal body weight. NIC dose was calculated based on predicted dam weight at the midpoint of gestation.

### 3.1. Endpoint Collection of Tissues, Plasma, Amniotic Fluid and Cecal Content

On GD19, or 13 days following pump surgery, the dams were sacrificed. Animals were anesthetized at a surgical plane with isoflurane (2.5% in 100% oxygen) and blood was collected from the aorta for detection of circulating plasma leptin and SCFA content. Following this, all animals were given an overdose of sodium pentobarbital (>200 mg/kg). Cecal tissue was collected from dams and flash frozen (−80 °C). Cecal fecal content was collected and snap frozen for determination of maternal microbiota composition and SCFA levels. As previously described [42], individual fetal/placental units were removed from the uterus and the amniotic fluid was collected separately from each fetal unit by puncturing the amniotic sac. Amniotic fluids were snap frozen for the measurement of leptin and SCFA levels. Fetuses were decapitated and blood was collected in BD Microtainer capillary blood tubes with serum separator for serum preparation. Fetal brains (with cortex removed), adrenals, kidneys and liver were flash frozen and stored in −80 °C for subsequent RNA isolation. Two to four of the fetal/placental unit tissues/dam were randomly assigned and dried and weighed for calculation of the fetal:placenta weight ratios as a measure of intrauterine growth restriction/stress [43]. The placental tissues from the remaining units (n = 8–10 per group) were snap frozen for analysis of placental gene expression [44,45,46]. Rat weights and numbers of fetal units per pregnant dam at the time of sacrifice were averaged within treatment groups and presented as mean ± SEM. Wet and dry fetal and placental weights and the ratio of fetal to placental dry weights were averaged within groups and presented as mean ± SEM.

### 3.2. Leptin Analysis in Maternal and Virgin Female Rat Serum, Fetal Serum and Amniotic Fluid

The leptin ELISA assay (KRC2281) was performed in accordance with the manufacturer’s instructions (Invitrogen). Serum samples (collected as described above) and amniotic fluid samples were diluted 1:3 in the assay dilution buffer and tested in triplicates. Standards and appropriate controls were prepared as per the assay instructions. Absorbance was determined at 415 nm using a BioRad iMark microplate absorbance reader and analyzed using Microplate Manager v6.0. Data were presented as median ± SEM.

### 3.3. Cecal SCFA Extraction and Analysis

Cecal samples were analyzed by Microbiome Insights (microbiomeinsights.com/, accessed on 25 June 2022, Vancouver, BC, Canada). As per the established methods [47], once received, materials were resuspended in MilliQ-grade H_2_O, and homogenized using MP Bio FastPrep, for 1 min at 4.0 m/s. Then, 5 M HCl was added to acidify the fecal suspensions to a final pH of 2.0. Acidified fecal suspensions were incubated and centrifuged at 10,000 rpm to separate the supernatant. Fecal supernatants were spiked with 2-ethylbutyric acid for a final concentration of 1 mM. Standard stock solutions of SCFAs were prepared and 2-ethylbutyric acid solution containing 12% formic acid was used as an internal standard stock solution control. Individual calibration curves were obtained for each SCFA using the standard SCFA mixture. Linearity of response for the standard acids was tested six times by seven or eight levels of concentrations, depending on the SCFA. SCFA levels were detected using gas chromatography (Thermo Trace 1310), coupled to a flame ionization detector (Thermo). The SCFA column used was a Thermo TG-WAXMS A GC Column of 30 m, 0.32 mm and 0.25 um. Regression equations of the standards were used to calculate sample concentration. Data were averaged within each group and presented as mean ± SEM. In addition, linear regression analysis was performed in GraphPad Prizm v8.0 to determine correlation between levels of cecal and plasma SCFAs. A slope of r^2^ ≥ 0.70 with a *p* value of <0.05 was considered as an indicator of high correlation with statistical significance.

### 3.4. 16S Bacterial Sequencing and Analysis

16S bacterial sequencing was performed in cecal samples from all groups (Microbiome Insights (microbiomeinsights.com/, accessed on 25 June 2022, Vancouver, BC, Canada) to determine cecal microbial composition in pregnant and virgin female rats. As per the company methods, DNA was extracted following MoBio’s instructions on a KingFisher robot. Bacterial 16S rRNA genes were PCR-amplified with dual-barcoded primers that targeted the variable V4 region (515F 5′-GTGCCAGCMGCCGCGGTAA-3′, and 806R 5′-GGACTACHVGGGTWTCTAAT-3′), as per the protocol of Kozich et al. [48]. Amplicons were then sequenced using Illumina MiSeq with the 300-bp paired-end kit (v.3). Sequences were denoised, taxonomically classified using Greengenes (v. 13_8) as the reference database, and clustered into 97%-similarity operational taxonomic units (OTUs) with the mothur software package (v. 1.39.5) [49] following the recommended procedure (https://www.mothur.org/wiki/MiSeq_SOP, accessed on 25 June 2022). The potential contamination was addressed by co-sequencing DNA amplified from specimens and from each four of the template-free controls and extraction kit reagents were processed the same way as the specimens. OTUs were considered putative contaminants (and were removed) if their mean abundance in the controls reached or exceeded 25% of their mean abundance in specimens. In order to assess the significance of differences in microbial composition within the different control (CON) and NIC groups, the QIIME RNA-Seq data were further analyzed using the linear discriminant analysis effect size (LEfSe) tool hosted on the online Galaxy application. The relative microbial abundance was plotted for each of the CON and NIC groups. The plotted data were then inputted through the LEfSe tool in Galaxy to produce a cladogram that visualizes significant differences in taxonomic branches of a phylogenetic tree represented within a certain treatment group. PCoA coordinates of taxonomic data were plotted using R with the ggplot2 package in RStudio. The PCOA plot was color-coded according to the data groups, with data ellipses illustrating the scope.

### 3.5. SCFA Extraction and Analysis in Maternal and Virgin Female Rat Serum and Fetal Amniotic Fluid

For SCFA extraction from serum and amniotic fluid, frozen samples were shipped overnight to Creative Proteomics (creative-proteomics.com, accessed on 25 June 2022, Shirley, NY, USA). As per the company’s methods, SCFA standards were dissolved in ethanol to generate standard concentration gradients from 0.001 mg/mL to 1000 mg/mL. Experimental samples were also resuspended in ethanol (containing 0.5% HCl, *v/v*) and centrifuged after ultrasonic treatment for 40 min. A volume of 1 µL was then injected through an Agilent DB-FFAP capillary column, fitted in an Agilent 7890–5977 gas chromatography–mass spectrometry system. Regression equations of the standards were used to calculate sample concentration. Data were averaged within each group and presented as mean ± SEM. In addition, linear regression analysis was performed in GraphPad Prizm v8.0 to determine correlation between levels of maternal cecal and plasma SCFAs, and maternal plasma and amniotic SCFAs separately. Slope r^2^ ≥ 0.70 with significance value of *p* < 0.05 was considered as an indicator of high correlation.

## 4. Gene Expression Analysis

Total RNA was isolated using TRIzol reagent (Invitrogen, Waltham, MA, USA), treated with DNase I, purified on RNeasy columns (Qiagen, Hilden, Germany), and then quantified with a NanoDrop Lite spectrophotometer (Thermo Fisher Scientific, Inc., Waltham, MA, USA). Equal concentrations of total RNA were reverse transcribed using oligo dT primers and the M-MLV (Moloney murine leukemia virus) Reverse Transcriptase kit (Invitrogen, Waltham, MA, USA) according to the manufacturer’s instructions. Quantitative qRT-PCR experiments were performed on a Roche LightCycler480 using Taqman hydrolysis probes (ThermoFisher, Waltham, MA, USA) and SYBR Green (Applied Biosystems, Waltham, MA, USA), with gene-specific primers. Standardization of the qRT-PCR data were performed with the endogenous reference gene *rGapdh* or ß-Actin. The samples were analyzed in triplicate with at least 5 biological replicates, and the fold change was calculated using the ∆∆Ct method as previously described and then represented as percent of control [50].

Genes evaluated in the placenta included IGF-2 (Rn00580426_m1), leptin (Rn00565158_m1) and hydroxysteroid 11-beta dehydrogenase 2 (11β-HSD2, Rn04341420_g1). These genes were evaluated for their known role in the placenta in fetal protection and normal development [51]. Genes evaluated in the adult female proximal colon were grouped into the following three categories: first, those genes involved in gut epithelial barrier health, including occludin (Ocln, Rn00580064_m1), mucin 3 (Muc3,), tumor necrosis factor (Tnfα, Rn01525859_g1), tryptophan hydroxylase 1 (Tph1, Rn01476867_m1). Genes involved in SCFA transport and select receptors, including free fatty acid receptor 2 (Ffar2, Rn02345824_s1), free fatty acid receptor 3 (Ffar3, Rn01457614_g1), glucagon (Gcg, Rn00562293_m1), glucagon-like peptide 1 receptor (Glp1r, Rn00562406_m1), and genes associated with specific transporters, including serotonin transporter (Sert), and sodium-coupled monocarboxylate transporter 1 (Slc5a8) [52] and the monocarboxylate transporter (MCT). Other genes evaluated in the hypothalamus and placenta showed no change (data not shown); genes and the corresponding primers are listed in Supplementary Table S1.

## 5. Statistical Analysis

Multivariate analysis was used to evaluate the effect of treatment on SCFA and leptin levels, gene expression, and microbial abundance in pregnant versus virgin females, using GraphPad (version 8.0, San Diego, CA, USA). One- and two-way ANOVA were used where appropriate, with post hoc comparisons when needed (Dunnett’s multiple comparisons test). The Mann–Whitney test was used to where appropriate. For the microbiome analysis, (Microbiome Insights) diversity was estimated with the Shannon index on raw OTU abundance tables after filtering out contaminants. The significance of diversity differences was tested with two-way ANOVA.To estimate beta diversity across the samples, OTUs that occurred with a count of less than 3 in at least 10% of the samples were eliminated and then computed using Bray–Curtis indices. We visualized beta diversity, emphasizing differences across samples, using principal coordinate analysis (PCoA) ordination. Variation in community structure was assessed with permutational multivariate analyses of variance (PERMANOVA), with treatment group as the main fixed factor and using 9999 permutations for significance testing. All microbiota analyses were conducted in the R environment. All results were considered significant at *p* ≤ 0.05.

## 6. Results

### 6.1. Impact of NIC on Body Weight, Fetal Weight, and Fetal Number

Tissues from 16 virgin and 13 pregnant females were used for data analysis. As shown in Table 1, at the time of sacrifice, overall body weight was greater in the pregnant versus virgin rats (*p* < 0.001; two-way ANOVA). NIC exposure did not change endpoint body weight within groups compared to the corresponding CONs (*p* > 0.25) and there was no interaction between NIC and the pregnant state (*p* > 0.72).

NIC during pregnancy did not alter the average number of fetuses per litter (15 ± 1 vs. 14 ± 1 fetuses/litter, CON vs. NIC, respectively; *p* > 0.6). Fetal wet body weight was greater than dry body weight (*p* < 0.001), but the average dry weight of the NIC exposed fetuses was not different from CONs (Table 1; *p* > 0.22). Similarly, wet placental weight was greater than dry weight (*p* < 0.001), but the average placental dry weight of the NIC exposed fetal units was also not different from CON units. Finally, the ratio of the dry fetal weight to placental weight tended to be lower in the NIC exposed compare to CON units; but this difference was not significant (*p* > 0.16). 

**Figure 1:**
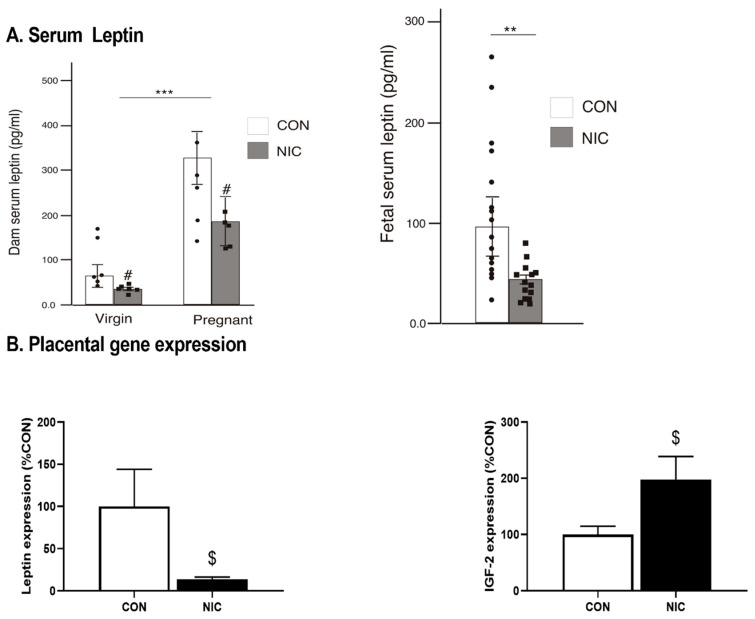
Serum and placental leptin levels were reduced, and placental IGF-2 gene expression elevated following 13 days of nicotine exposure (NIC; 6 mg/kg/day) compared to controls (CON). (**A**) Serum leptin levels measured in adult female (left graph; n = 6–8/group) and fetuses (right graph; n = 12/group). (**B**) Placental gene expression for leptin (left graph) and IGF-2 (right graph; n = 8–11/group). Values are median ± SEM (**A**) and mean ± SEM (**B**); ** and *** indicate *p* < 0.01 and *p* < 0.001 respectively, between virgin and pregnant groups combined; # indicates *p* < 0.019 between NIC vs. CON in virgin and pregnant females combined (two-way ANOVA). $ indicates *p* < 0.05 between CON and NIC (Mann–Whitney test).

Parallel to the rise in body weight, serum leptin levels during pregnancy increased when compared to the virgin state (Figure 1A, *left graph*; *p* < 0.02; two-way ANOVA). Although we detected no effect on the weight, NIC reduced adult female serum leptin levels compared to CONs (*p* < 0.0001), but there was no interaction with pregnancy status (*p* > 0.17). Despite the increase in serum leptin in the dams during pregnancy in both groups, NIC exposure reduced fetal serum leptin levels compared to CONs (Figure 1A, *right graph*; *p* < 0.025). This NIC-associated drop in fetal serum leptin was paralleled by a reduction in leptin gene expression (Figure 1B, *left graph*; *p* < 0.05;) and an upregulation of placental *Igf2* gene expression (Figure 1B, *right graph*; *p* < 0.04) in the placentas from the NIC fetal units compared to CONs. There was no difference in *11ß-Hsd2* expression in the placenta between groups (100 ± 22 vs. 134.3 ± 30 percent of CON, CON vs. NIC: *p* > 0.37). Similarly, no significant difference was observed in the placental or hypothalamus expression of other genes associated with growth, epigenetic regulation and cardiovascular development (genes and corresponding primers are listed in Supplementary Table S1). 

### 6.2. Effect of NIC and Pregnancy on the Female Gut Microbiome

**Figure 2:**
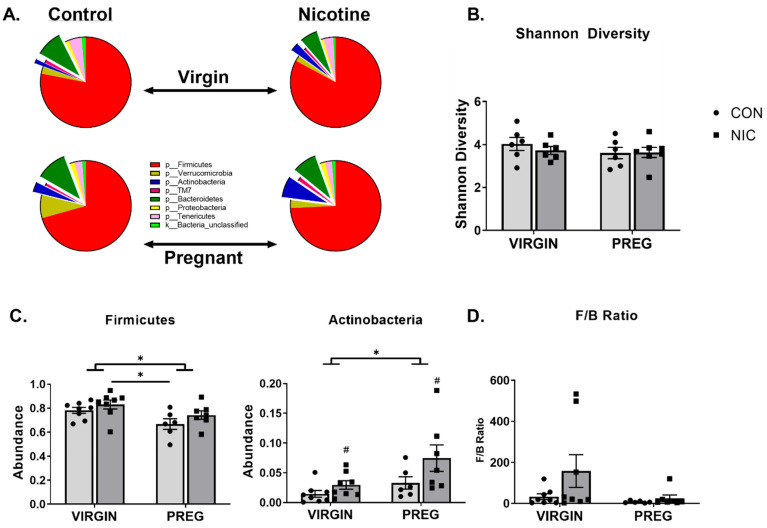
Nicotine (NIC) exposure shifts cecal bacterial abundance in virgin versus pregnant (preg; gestational day 19) Sprague Dawley rats. (**A**) Average proportion of bacteria within each phylum identified. (**B**) Gut microbial diversity as measured by Shannon diversity index. (**C**) Two-way ANOVA identified significant shifts in bacteria in phyla Firmicutes and Actinobacteria in pregnancy (*p* < 0.02). NIC also elevated the proportion of bacteria in p_Actinobacteria, independent of pregnancy status (*p* < 0.04). (**D**) The ratio of bacteria in p_Firmicutes to p_Bacteriodetes or F/B ratio was unchanged by pregnancy or NIC exposure. Values are mean ± SEM; Data were analyzed by two-way ANOVA with multiple comparisons. * (handle bars) indicates *p* < 0.02 between virgin and pregnant groups combined; # indicates *p* < 0.04 between NIC vs. CON in virgin and pregnant females combined; * (straight line) indicates *p* < 0.05 between treatment groups. n = 6–8 samples per group.

Cecal bacterial abundance and distribution were compared between virgin CON (n = 8), virgin NIC (n = 8), pregnant CON (n = 6) and pregnant NIC (n = 7) dams. Seven major phyla were identified in the female cecal gut. These included P_Firmicutes, p_Bacteriodetes, p_Tenericutes, p_Verrucomicrobia, p_Actinobacteria, p_Proteobacteria, and p_TM7. The averaged abundance of bacteria in the different phyla for each treatment group is shown in Figure 2A. The general trend was for pregnancy to reduce the abundance of p_Firmicutes (red), and NIC generally elevated the abundance of the p_Actinobacteria (blue). Analysis of the alpha diversity of bacteria or Shannon diversity index (Figure 2B) identified a trend for a decrease in diversity in pregnancy (*p* < 0.06, two-way ANOVA), but there was no change in the general alpha diversity associated with NIC (*p* > 0.69).

Further analysis of the relative bacterial abundance within each phylum identified that in the pregnant state, there was a decrease in the relative abundance of bacteria in p_Firmicutes compared to the virgin state (*p* < 0.009; Figure 2A,C). In contrast, pregnancy increased the relative abundance of bacteria in the p_Actinobacteria compared to the virgin state (Figure 2C, *middle panel*; *p* < 0.02). Relative to the virgin state, during pregnancy, there was also a trend for an increase in the overall abundance of p_Verrucomicrobia (Figure 2A; *p* < 0.08). The only independent effect of NIC was observed in the abundance of p_Actinobacteria, where NIC induced an increase in the abundance of this phylum relative to CON (*p* < 0.04), but there was no interaction between NIC and state of pregnancy (*p* > 0.30). There was no significant effect of pregnancy or NIC on bacterial abundance in the remaining phyla (*p* > 0.09). 

In addition to the changes in individual phylum, as an indicator of overall gut microbiota health, the ratio of bacterial abundance in the p_Firmicutes versus p_Bacteriodetes was calculated. As shown in Figure 2D, the F/B ratio tended to be lower during pregnancy compared to the virgin state (*p* = 0.09; two-way ANOVA) and NIC appeared to generally increase the F/B ratio (*p* = 0.13), but these changes were not significant, likely due to high variability in the ratios. 

**Figure 3:**
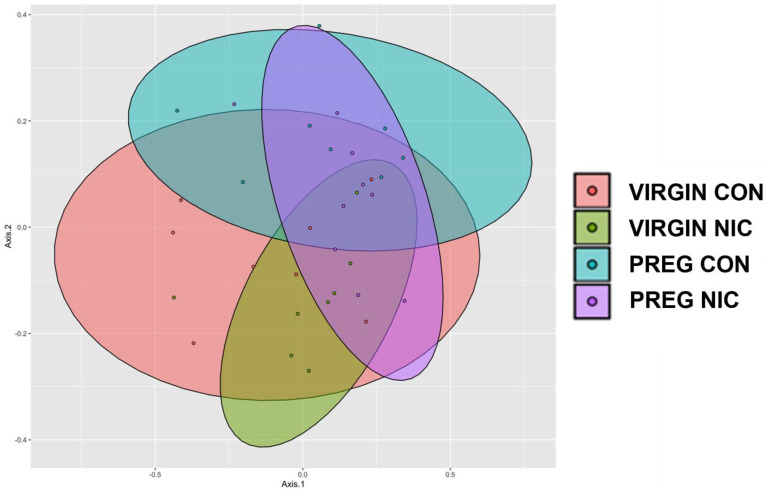
Principle coordinate analysis (PCoA) comparing the impact of nicotine exposure in virgin and pregnant female SD rats. NIC generally narrowed the clusters in both groups compared to CON, with the greatest shift during pregnancy. Data are based on 16s sequencing, n = 6–8 samples per group.

The diversity between samples (Shannon beta diversity) was quantified using the Bray–Curtis dissimilarity method (Microbiome Insights; Bray and Curtis 1957, Figure 2B). Permutational analysis identified significant differences between the virgin and pregnant groups only (Figure 2A, *p* < 0.05). Although there was considerable overlap between all groups, there were distinct regions of non-overlap between the virgin NICs and pregnant CONs (olive vs. aqua colors, respectively, *p* = 0.054), virgin NICs and pregnant NICs (olive vs. purple colors, respectively, *p* = 0.054) and virgin CONs and pregnant NICs (orange vs. purple colors, respectively, *p* = 0.054). To display the differences between the groups, an unweighted principal coordinate analysis (PCoA) was also performed (Figure 3). 

**Figure 4:**
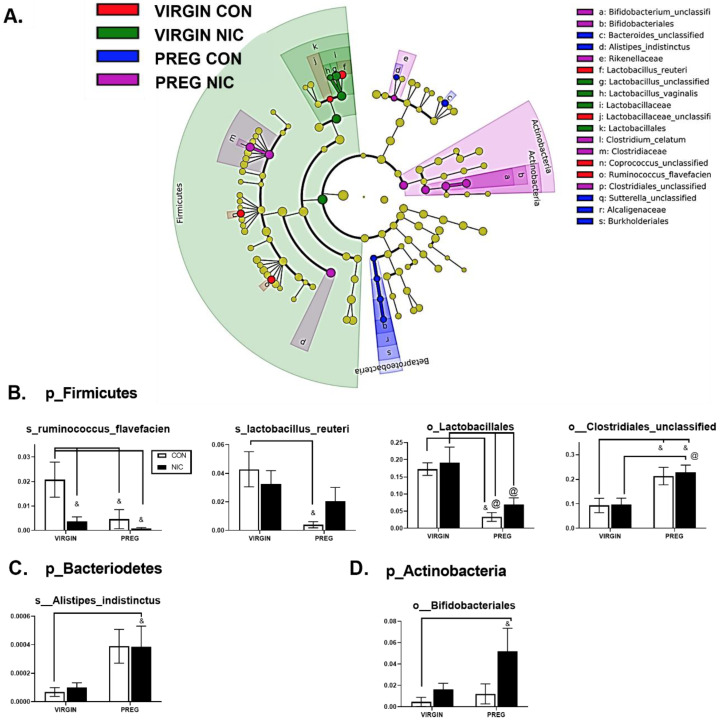
Nicotine exposure in rodent pregnancy induces selective changes in cecal bacterial abundance in Firmicutes, Bacteriodetes, and Actinobacteria phyla. (**A**) Taxonomic cladogram generated in Galaxy demonstrating shifts in taxonomic groups following nicotine exposure in the virgin versus pregnant rats (P). Each circle represents a bacterial taxon, with its diameter proportional to the taxon’s relative abundance. Yellow circles indicate no change. Colored circles indicate significant changes in abundance relative to other groups. Red—virgin controls; green—virgin nicotine exposed; blue—pregnant controls and purple—pregnant-nicotine exposed data. (**B**–**D**) Two-way ANOVA analysis with multiple comparisons was performed and revealed significant differences in order (o_) or species (s_) between groups in the phylum Firmicutes, Bacteriodetes and Actinobactera, as annotated. Values are mean ± SEM; & indicates *p* < 0.05 between CON in virgin and other groups as indicated; @ indicates *p* < 0.02 between virgin nicotine versus other groups indicated. n = 6–8 samples per group.

Specific bacterial taxonomic shifts were further highlighted using linear discriminate analysis (LDA) of size effect in Galaxy (Figure 4A). Examples of specific changes between the treatment groups are shown in Figure 4B. A decline in bacterial abundances of s_Ruminococcus_Flavefacien, s_Lactobacillus_Reuteri and o_Lactobacillales were observed during pregnancy compared to the virgin state (*p* < 0.04; two-way ANOVA). For s_Ruminococcus_Flavefacien, there was also a reduction in abundance associated with NICs compared to virgin CONs (*p* < 0.009). In o_Lactobacillales, there was a decline in abundance during pregnancy in both NIC and CON groups, compared to virgin NIC (*p* < 0.03). In contrast, there was an increase in abundance of o_Clostridiales during pregnancy relative to the virgin CON and NIC groups (*p* < 0.04). As shown in Figure 4C,D, bacterial shifts from the virgin to pregnant state were associated with increases in the general abundance of p_Bacteriodetes and p_Actinobacteria. In p_Bacteriodetes, an increase in bacterial abundance in s_Alistipes_indistinctus was identified between the NIC pregnant group compared to the virgin CON group (*p* < 0.05) and there was a trend for an increase in the CON pregnant group also (*p* = 0.057). Similarly, in p_Actinobacteria (Figure 4D), the increase in abundance of o_Bifidobacteriales was identified between the NIC pregnant group compared to virgin CONs (*p* < 0.05). 

**Figure 5:**
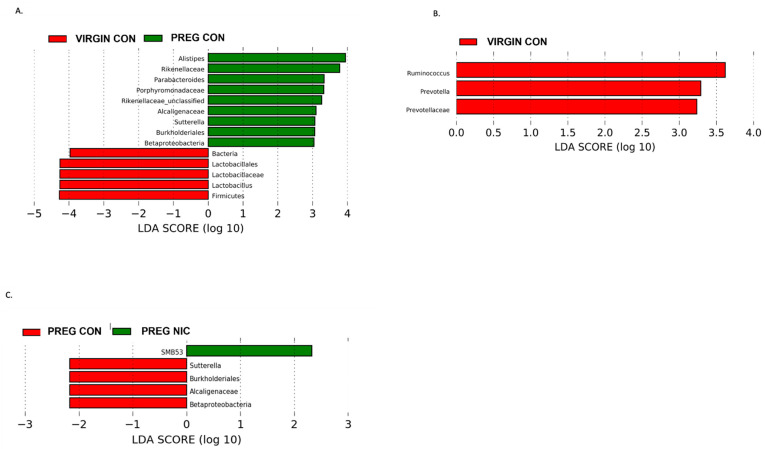
Effect of pregnancy and nicotine on cecal bacterial abundance and composition in female SD rats. LDA analysis of cecal bacterial taxa demonstrates significant shifts in taxonomic groups during pregnancy alone (in (**A**) red = virgin control; green = pregnant control), during s.c. exposure to nicotine in female SD rats (in (**B**) red = virgin control, green = virgin nicotine), and during pregnancy with exposure to s.c. nicotine (in (**C**) red = pregnant control, green = pregnant nicotine) in SD dams. Each color represents a more abundant bacterial taxon relative to the other group. A marked decrease in Proteobacteria and specifically Betaproteobacteria in the PREG NIC vs. PREG CON group is shown (in (**C**)), while an elevated abundance in Betaproteobacteria may be important in normal rodent pregnancy (in (**A**)). Data are based on 16s sequencing analysis, n = 6–8 per group.

In Figure 5, we performed additional LDA analysis to compare changes in cecal bacterial taxa with pregnancy alone (A), and to elucidate the effects of NIC infusion in virgin females (B) and in pregnant dams (C) alone. In pregnant dams (C), we observed a marked decrease in Proteobacteria and specifically Betaproteobacteria with NIC infusion (P_NIC), which may be important in normal pregnancy (P_CON), as also illustrated in A. In virgin dams (B), we observed an abundance of Riminococcus, Prevotella and Prevotellaceae in the CON vs. NIC groups, suggesting a reduction in these bacteria with NIC in virgin female rats.

### 6.3. Effect of NIC and Pregnancy on SCFAs in the Female SD Rats

**Figure 6:**
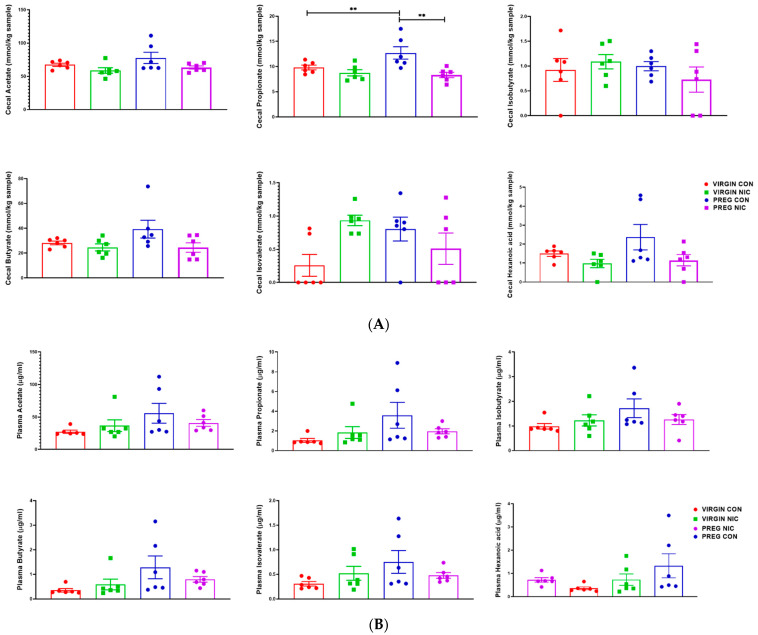

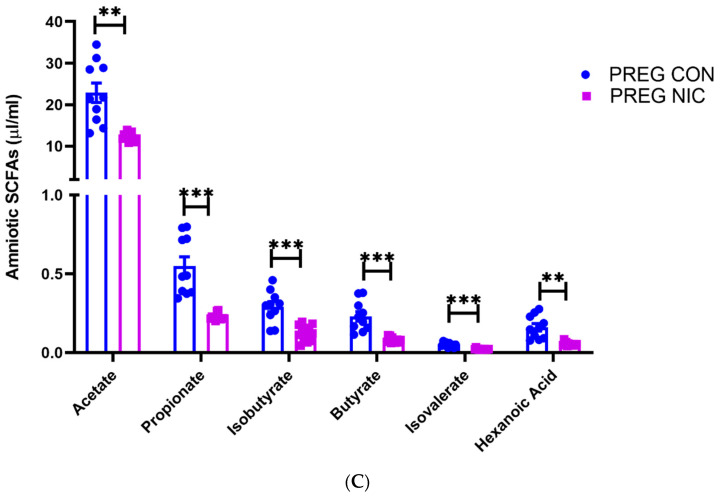
In (**A**), propionate increased during pregnancy in control (PREG CON), while nicotine (PREG NIC) exposure reduced cecal propionate levels in pregnant dams. All other cecal SCFAs were unchanged by pregnancy or NIC exposure. In (**B**), no significant differences were observed in circulating SCFAs between the groups. However, in (**C**), all measured SCFAs were significantly reduced in amniotic fluid collected from PREG NIC group. Values expressed as mean ± SEM; Data were analyzed using one-way ANOVA with multiple comparisons. ** *p* < 0.01, *** *p* < 0.001, n = 6–10 per group.

**Figure 7:**
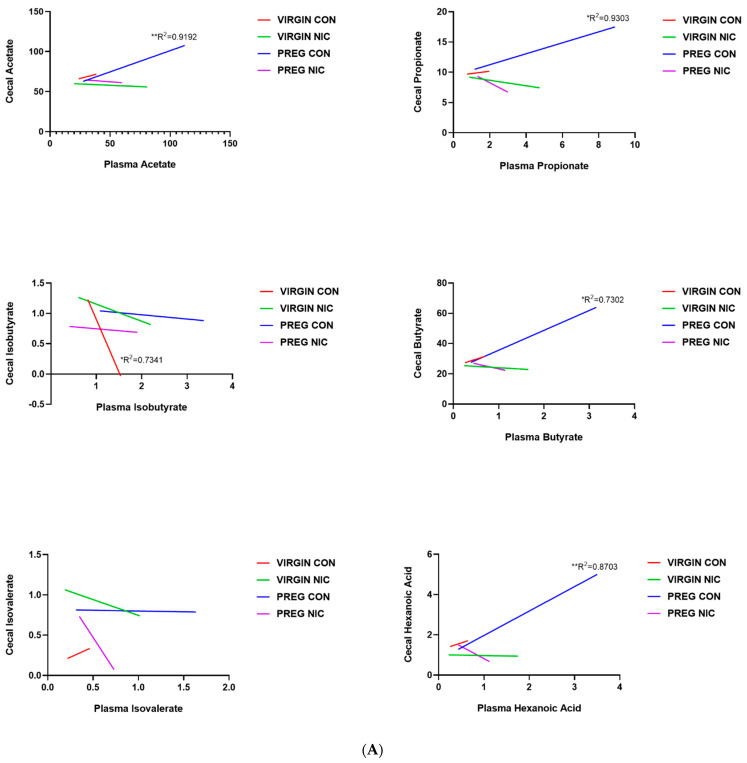

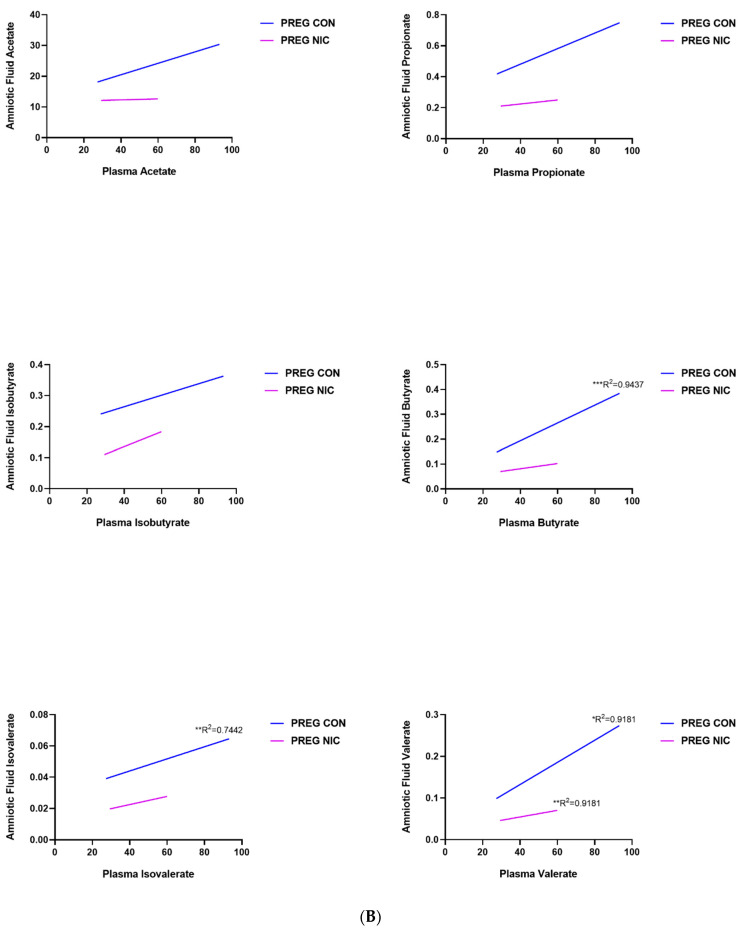
Relationship between cecal, plasma and amniotic fluid SCFAs in pregnant dams on nicotine. In (**A**), a positive correlation between cecal and circulating (plasma) acetate, propionate, butyrate and hexanoic acid was observed in pregnant control (PREG CON) dams alone. In (**B**), a positive correlation between circulating (plasma) and amniotic fluid levels of all measured SCFAs was observed in some samples from PREG CON dams. Linear regression analysis was performed, * *p* < 0.05, ** *p* < 0.01, *** *p* < 0.001 from zero; n = 8–10 samples per group.

An important function of bacteria in the gut is to metabolize food products that are not absorbed in the small intestine, generating SCFAs. The SCFAs are the utilized by the GI tract and transported into circulation. From there, the SCFAs may be delivered to the fetal units via amniotic fluid. Thus, we measured the changes in several SCFAs in the cecal content (Figure 6A) and in the plasma (Figure 6B) of virgin females and pregnant dams, as well as in the fetal amniotic fluid from pregnant dams (Figure 6C) infused with a vehicle control (CON) or NIC. We further performed a correlation analysis to investigate whether there is a correlation between SCFAs levels in maternal cecum and plasma, indicative of maternal ‘handling’ of SCFAs in pregnancy and with NIC infusion. In addition, a correlation analysis was performed between the SCFA levels in maternal plasma and amniotic fluid, to investigate the effects of NIC on the availability of select SCFAs to the fetal units during pregnancy. We observed that pregnancy alone elevated propionate levels in the cecum (blue bar, Figure 6A, *p* < 0.01; two-way ANOVA), while NIC significantly reduced cecal propionate levels in pregnant dams (purple bar, Figure 6A, *p* < 0.01; two-way ANOVA). Pregnancy tended to increase, while NIC tended to decrease, the plasma levels of all measured SCFAs, apart from hexanoic acid (Figure 6B), although this did not reach statistical significance. However, the levels of all measured SCFAs were significantly reduced in the amniotic fluid of pregnant dams with NIC infusion (P_NIC, Figure 6C, two-way ANOVA). To further explore the effects of NIC in pregnancy on the relationship between fecal, plasma and amniotic fluid SCFAs, we performed linear regression (Figure 7). We observed a significant positive correlation (R^2^ > 0.70) between cecal and plasma levels of acetate, propionate butyrate and hexanoic acid in pregnant controls (P_CON, Figure 7A), and a negative correlation betweencecal and plasma isobutyrate in virgin controls (NP_CON, Figure 7A). In addition, we observed significant positive correlation between plasma and amniotic butyrate, isovalerateand valerate in pregnant controls (P_CON, Figure 7B), while a positive correlation correlation between plasma and amniotic valerate was also observed in pregnant DAMS following nicotine (P_NIC, Figure 7B). Hexanoic acid was undetectable in the amniotic fluid in both groups. 

### 6.4. Pregnancy and NIC-Induced Changes in Cecal Tissue Relative Gene Expression

**Figure 8:**
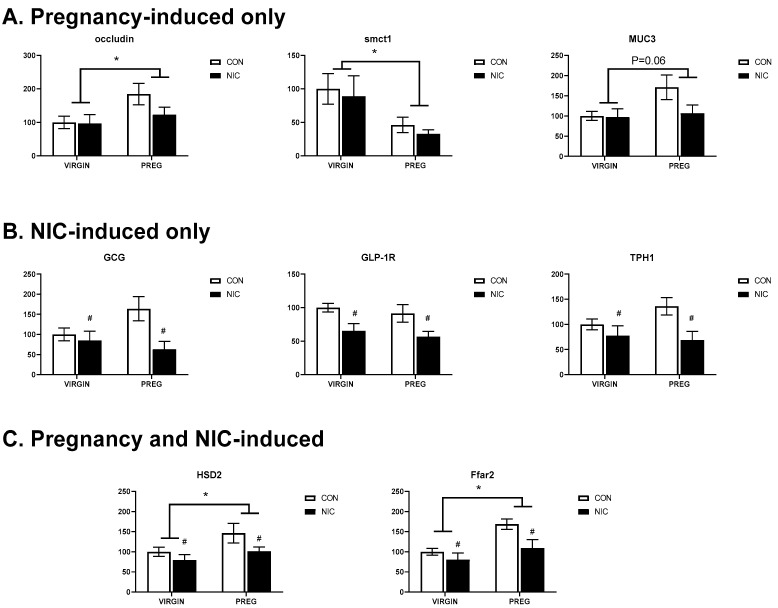
Pregnancy and NIC alter gene expression in cecal tissue. (**A**) Pregnancy (PREG) alone upregulates expression of occluding and mucin 3 (muc3) and downregulates expression of sodium-coupled monocarboxylate transporter 1 (smctl), the lactate transporter. (**B**) NIC downregulated expression of genes associated with glucagon (Gcg), glucagon-like protein type 1 receptor (Glp-1r) and tryptophan hydroxylase 1 (Tph1), the gene associated with serotonin synthesis. (**C**) Pregnancy upregulated the expression of 11-beta-dehydrogenase isozyme 2 (Hsd2), the gene associated with inactivation in glucocorticoids and the free-fatty acid receptor 2 (Ffar2), the G-protein-coupled receptor for SCFAs. The expression of both genes were down-regulated by nicotine exposure. Values are mean ± SEM; Data were analyzed using two-way ANOVA with multiple comparisons. * indicates *p* < 0.02 between virgin and pregnant groups combined; # indicates *p* < 0.04 between NIC vs. CON in virgin and pregnant females combined. n = 6–8 samples per group.

Changes in SCFA transporters and other indicators of gut function were quantified via gene expression analysis. As shown in Figure 8A, changes in relative gene expression in the female cecal tissue that were primarily altered in response to pregnancy included an upregulation of the tight junction regulator occludin (*p* < 0.04; two-way ANOVA), down regulation of the sodium-coupled monocarboxylate transporter 1 (*Smct1* or *Slc5A8*; *p* < 0.02) linked to SCFA transport, and a strong trend for upregulation of mucin 3 (*Muc3*; *p* < 0.06) linked to gut epithelial lining protection.

Figure 8B shows the expression of those genes that were primarily altered by NIC. Interestingly, in all three instances, NIC induced a downregulation of gene expression, including GCG or preproglucagon (*p* < 0.019; two-way ANOVA), GLP-1, a peptide hormone receptor in the gut that plays important roles in regulating appetite and blood sugar levels and has been linked to regulating both gut motility and inflammation (*p* < 0.003), and TPH1, the rate limiting enzyme involved in the production of serotonin by enterochromaffin cells in the gut and linked to the maintenance of gut health, appetite, absorption and inflammatory responses (*p* < 0.015). Finally, Figure 8C shows the three genes altered by both pregnancy and NIC. Included in this group was 11-beta-HSD2, which is involved in metabolizing glucocorticoids and was upregulated by pregnancy (*p* < 0.043; two-way ANOVA) but was downregulated by NIC (*p* < 0.05). The exact role of HSD2 in the gut is not understood, but may be linked to increased metabolism of cortisol, potentially modulating fetal exposure to stress hormones during pregnancy, similar to its role in the placenta. *Ffar2*, or free fatty acid receptor 2 (*Gpr43*), expression was also upregulated by pregnancy (*p* < 0.005) but downregulated by NIC (*p* < 0.0018). This pattern is similar to that observed for its primary ligands, the cecal SCFAs acetate and propionate. Additional genes were evaluated but did not show any change in pregnancy and/or NIC-included *Tnf* (*p* > 0.42), lactate transporter *Slc16a1* (*p* > 0.08), *Sert1* (*p* > 0.1), and *Ffar3* (*p* > 0.38).

## 7. Discussion

It has been hypothesized that certain adult conditions, such as obesity, cardiovascular disease, and neurological disorders, may originate in utero in response to different stressors, leading to changes in gene expression [53,54]. Considering the role of gut microbiota and their select metabolites, such as the SCFAs, in the regulation of prenatal and postnatal health, we undertook the current study to determine whether exposure to nicotine during pregnancy was linked to changes in the maternal gut microbiome and consequently to changes in circulating and amniotic SCFA levels and select genes. We obtained the following findings: (i) exposure to NIC reduced serum leptin levels in pregnant dams and their fetal units, which was associated with a decrease in relative expression levels in the placental leptin gene; (ii) pregnancy alone caused a significant reduction in the abundance of p_Firmicutes, and more specifically Ruminoccocus Flavefacien, which was also decreased by exposure to nicotine in both virgins and pregnant dams. Moreover, nicotine exposure during pregnancy significantly increases p_Actinobacteria of the order of Bifidobacteriales, while a reduction in Betaproteobacteria was observed in pregnant dams following exposure to nicotine. (iii) Changes in gut bacterial composition following NIC exposure were coupled with a general downregulation of cecal SCFA content and, more specifically, propionate. This was paralleled by changes in gene expression in the cecal tissue of dams, including both a general down regulation of genes linked to energy regulation, serotonin synthesis, and SCFA receptors. (iv) The NIC-induced drop in cecal propionate was associated with a similar trend in the serum of pregnant dams, and a significant drop in propionate, in addition to all other SCFAs tested in the amniotic fluid of nicotine-exposed fetal units. In the pregnant controls, we observed a significant strong positive correlation between cecal and plasma levels of acetate, propionate, butyrate and hexanoic acid, and exposure to nicotine eliminated this effect. This correlation was not present in the virgin controls, suggesting that pregnancy may shift the emphasis on transport of SCFAs to circulation, where it may be more available to the fetal units. In line with this, a strong significant positive correlation between maternal circulation and amniotic fluid in pregnant dams was observed for butyrate, valerate and isovalerate, while exposure to nicotine eliminated this effect in all but valerate. These data suggest that exposure to nicotine during rodent pregnancy alters maternal gut microbiota composition, which then may lead to changes in the availability of bacterial SCFAs and particularly butyrate and isovalerate to fetal units *in utero*.

We determined that pregnancy itself produces shifts in the gut microbiota. An overall reduction in Firmicutes, reported producers of propionate, was observed in pregnant dams, while NIC further reduced Ruminoccocus Flavefacien in pregnant dams. A reduction in Ruminoccocus Flavefacien has previously been associated with obesity and hypertension in rodents and in obese individuals [55,56]. In contrast, increased levels of Actinobacteria, also associated with obesity (reviewed in [57]), and specifically Bifidobacteriales, have been found in NIC-exposed pregnant dams, suggesting a microbiota link between in utero NIC exposure and obesity that develops later in the life of NIC-exposed offspring. Interestingly, reduced Actinobacteria, and specifically Bifidobacterium, have been associated with rodent hypertension [58], in contrast to our observations in NIC-exposed dams. While we did not measure blood pressure in our dams in this study, further studies investigating this are warranted. Moreover, decreased Betaproteobacteria observed in pregnant NIC compared to pregnant CON dams has previously been associated with inflammation [59], and type 2 diabetes [60]. Thus, changes in maternal microbiota may provide biomarkers of nicotine-induced cardiometabolic disease in the offspring, as previously suggested.

One recognized mechanism through which shifts in the maternal microbiome may influence fetal development is by a change in the production of metabolites, particularly SCFAs through microbial fermentation. SCFAs are the main metabolites produced by the microbiota and are considered an important communication tool between the bacteria and the host. During pregnancy, SCFAs act like signaling molecules to regulate bodily energy homeostasis, and propionate can act via its receptor FFAR2 (GRP43) to determine the development and metabolic programming of the fetus in pregnant women [61,62]. In addition, SCFAs could influence cell function through local modification of the Krebs cycle via conversion to acetyl-CoA in different cell subtypes [63] or through epigenetic modification of gene expression [64]. Our data show that in utero exposure to NIC reduces relative expression levels of *Ffar2*, and reduced cecal maternal and fetal amniotic fluid propionate, among the other SCFAs measured. While further studies need to be conducted, this reduction in amniotic SCFAs may present a mechanism behind the cardiometabolic dysfunction observed in the offspring following in utero NIC exposure [65,66].

We also observed a correlation between the circulating levels of select maternal SCFAs and those in the amniotic fluid of fetal units. SCFAs transported from the gut into the circulation can cross the placental barrier, modifying fetal development [19,67]. In normal pregnancy, the placenta plays a critical role in providing a protective barrier for the fetus by limiting or eliminating exposure to substances in the maternal circulation [68]. Recent evidence from our laboratories, however, suggest that nicotine exposure weakens the protective effect of placental barrier [42]. Thus, maternal smoking may induce significant changes in the maternal microbiome and circulating SCFA levels, which the fetus is then exposed to via changes in placental integrity [69,70]. In the gut, SCFAs can activate GPCRs on epithelial cells to modulate hormone release (i.e., serotonin, glucagon-like peptide 1 (GLP-1), etc.) [71], thereby indirectly regulating metabolic function. In our study, we observed significantly reduced relative expression levels of glucagon (*Gcg*), Glp1 receptor (*Glp1r*) and tryptophan hydroxylase 1 (*Tph1*) in the cecal tissue from CON and pregnant dams following NIC exposure. Considering the role of GCG, GLP-1 and serotonin in appetite, absorption and metabolism, it is possible that the NIC effects on these genes are mediated via the modulation of SCFA production in the gut. For example, offspring born to obese mothers on a high fat diet during pregnancy, which is associated with an imbalance of gut bacteria or gut dysbiosis and significant changes in circulating SCFA levels [72,73], have an increased incidence of obesity. This offspring obesity has been linked to changes in both offspring gut microbiome and hypothalamic gene expression associated with metabolic regulation, including the leptin system [29,30,74]. In circulation, SCFAs have multiple functions, including regulation of cardiovascular health (reviewed in [25,26]) and modulation of leptin release from adipose tissue cells via GPCR activation [27]. Thus, as a result of maternal dysbiosis and sustained changes in SCFA levels, developmental circuits involved in regulating obesity, and possibly blood pressure, may be significantly altered [28,29,30].

Although we did not directly measure GLP-1 or serotonin, our data show a significant decrease in circulating leptin in both the pregnant dams and their offspring following NIC exposure. Leptin is an adipokine that is released by both maternal adipose tissue and the placenta [75] and its role as a biomarker of obesity has been well studied. Normally, peripheral release of leptin inhibits food intake via activation of central leptin receptors. In the face of sustained leptin release, however, the central receptors downregulate, which leads to leptin resistance, over-eating and obesity. Interestingly, circulating SCFAs have also been reported to stimulate the release of leptin via GPCR activation in peripheral fat cells [27]. In infants, a positive correlation between birth weight and cord leptin levels has been noted [76]. Similarly, when compared to the virgin state, elevations in serum leptin have been noted during pregnancy in rats, with a peak in serum levels in late gestation [75]. The role of leptin in pregnancy is not fully established, but is thought to be involved in fetal growth, nutrient transport, and the development of leptin receptors in the brain [77]. Serum leptin levels become dysregulated in fetal growth restriction, obesity, and pre-eclampsia, all conditions that elevate risk for life-long disease [78]. In adults, leptin levels have been reported to fluctuate in response to cigarette smoking, although mixed results have been reported [79,80,81]. During pregnancy, however, several studies have consistently identified that either the number of cigarettes smoked, or pregnancy plasma cotinine levels, negatively correlated with maternal or cord leptin levels [82,83,84].

In the current study, NIC exposure was shown to decrease serum leptin levels in both virgin and pregnant females. We also showed that the NIC-induced decline in maternal leptin was paralleled by a decline in fetal leptin and placental gene expression, supporting a negative correlation between fetal NIC exposure and leptin levels during gestation. Changes in NIC exposure and leptin release are thought to be indirect. For example, in one study, it was shown that cigarette smoking significantly reduced plasma leptin levels, while simultaneously increasing plasma adrenaline levels, independent of any direct effect of NIC exposure on leptin expression in vitro [85]. Since catecholamines are known to decrease leptin release, this suggests that signals secondary to NIC exposure may contribute to altered leptin release during pregnancy [86]. Alternatively, leptin release is stimulated by glucocorticoids, and in pregnant Wistar rats, NIC exposure during pregnancy has been shown to elevate fetal corticosterone exposure, paralleled by a reduction in placental 11b-HSD2 expression [87]. In the current study, we did not measure plasma glucocorticoids, but we did measure cecal and placental 11β-HSD2 expression and found an increase in cecal expression but no difference in placental expression between groups, suggesting that there was potentially no difference in circulating glucocorticoids in the dams and fetal units. It is possible that these differences are due to the differences in NIC treatments (subcutaneous injections twice a day vs. continuous exposure in our study), since acute NIC exposure has been shown to elevate cortisol levels, while continuous exposure may not [88].

The decline in leptin observed in the current study was coupled to an upregulation of placental *Igf2* gene expression in the pregnant NIC exposed dams. *Igf2* is known to play a role in fetal-placental growth and IGF levels. IGF2 levels rise during pregnancy but fall just prior to birth, similar to leptin [89]. In rodents, there is evidence that NIC exposure during pregnancy can prevent the fall in *Igf2* that normally occurs during late gestation. Although we did not monitor the changes in *Igf2* expression throughout gestation, this pattern would fit with the elevated placental *Igf2* gene expression observed here in late gestation (GD19 out of 21). Interestingly, the effects of a restricted diet on *Igf2* expression appear to differ between the species; similar pattern of elevated *Igf2* has been reported in pregnant baboons with a moderately restricted diet [90], while reduced placental levels of *Igf2* have been reported in guinea pigs in response to a similarly restricted diet during pregnancy [91], and no difference in *Igf2* levels was reported in mice on a slightly less restricted diet [92]. Thus, because both leptin and *Igf2* levels fluctuate throughout gestation, further investigations into the impact of NIC exposure versus poor nutrition need to include measurements at multiple time points during gestation, before parallels can be drawn or links to adult diseases, such as obesity and hypertension, can be determined [78]. Since leptin is involved in the regulation of metabolism and shifts in placental *Igf2* have been linked to states of poor nutrition or fetal stress (reviewed in [93,94]), our results suggest, for the first time, that the predisposition for the offspring of smokers to develop obesity and cardiovascular disease later in life is linked to NIC-induced signals that mimic maternal poor nutrition. Studies of undernutrition during pregnancy in both humans and animal models demonstrate that there is a general reduction in offspring body weight at birth and a predisposition for offspring to develop obesity, impaired glucose tolerance, and indices of cardiovascular disease, with variations in phenotypes, depending upon the time during gestation of exposure [95]. In the current study, we did not observe any significant drop in fetal body weight or placental weight or the ratio of the two, suggesting that NIC exposure is perhaps a milder stressor than undernutrition. In this respect, the study from a maternity hospital in Ireland reported no difference for birthweight between babies born to women who self-reported vaping and those who neither vaped nor smoked during the last trimester of their pregnancy [96].

It is important to note, however, that during the last few days of gestation in the rodents, there is a burst in growth [75,97]. Furthermore, prenatal NIC exposure in rats has been reported by other investigators to induce a reduction in birth weight [98,99]. Thus, it is possible that if gestation had been allowed to go to completion, a reduction in birth weight would also have been identified in the current study.

In the present study, we also evaluated changes in gene expression in the cecum of pregnant dams and virgin females. Pregnancy alone induced an upregulation of expression of *occludin* and *Muc3*, both suggesting a shift toward a healthier gut lining. On the contrary, there was a downregulation of *Smct1* gene expression linked to pregnancy. The *Smct1* gene is linked to transport of SCFAs and considering an increase in cecal propionic acid in pregnancy, down regulation of this transporter may represent a compensatory mechanism to maintain constant SCFA levels in circulation during pregnancy.

Although these are informative data, we are aware of the limitations of our model in that the delivery of nicotine during smoking is not typically subcutaneous. Future experiments should evaluate the effect of inhaled nicotine and other substances contained in tobacco products.

## 8. Conclusions

Exposure to nicotine during pregnancy alters maternal gut microbiota. This is closely linked to a reduction in select maternal SCFAs. This decrease is associated with reduced levels of SCFAs in the amniotic fluid of fetal units exposed to nicotine in utero. Furthermore, nicotine exposure reduced circulating leptin levels in mothers and their respective fetal units, as well as placental expression of leptin, but increased placental expression of *Igf2*, suggesting an association between the alterations in maternal microbiota and expression of metabolism-related genes. Further studies are needed to fully understand the mechanisms of action of SCFAs in pregnancy and elucidate the possible epigenetic mechanisms behind alterations in select genes that may affect postpartum development and long-term homeostasis.

## Figures and Tables

**Table 1:** Weight by group.

	CON	NIC
Virgin body weight	216 ± 10.6 (n = 8)	227 ± 18.1 (n = 8)
Pregnant body weight	308 ± 11.5 (n = 6)	328 ± 10.6 (n = 7)
Placental dry weight	0.091 ± 0.008 (n = 16)	0.086 ± 0.004 (n = 16)
Fetus dry weight	0.41 ± 0.05 (n = 14)	0.34 ± 0.06 (n = 15)
Dry fetus/placental weight	4.36 ± 0.36 (n = 13)	3.82 ± 0.20 (n = 15)

## Data Availability

The data presented in this study are available upon request from the corresponding author. The data are not publicly available as they are undergoing a peer-review process at Metabolites.

## Supplementary Materials

The following are available online at https://www.mdpi.com/article/10.3390/metabo12080735/s1, Table S1: SYBR Green Real Time PCR Primers [100,101,102,103,104,105,106,107,108,109,110,111,112,113,114,115,116,117,118,119,120].
