# Supplementary material for: Nicotine Exposure during Rodent Pregnancy Alters the Composition of Maternal Gut Microbiota and Abundance of Maternal and Amniotic Short Chain Fatty Acids
Source: Metabolites. 2022 Aug 9;12(8):735. doi: 10.3390/metabo12080735 (PMC9414314; doi:10.3390/metabo12080735)
Supplement: Supplementary file 1 [file metabolites-12-00735-s001.zip › metabolites-1813213-supplementary.pdf]

Table S1. SYBR Green Real Time PCR Primers.

	Gene Name	Forward	Reverse	NCBI RefSeq	Tissue Tested	P Value	Ref
1	Ace	TTGTCTGTCACTGGAGCCTGA	CCACACCCAAAGCAATTCTTC	NM_012544.1	hypothalamus	0.07<br>0.12(m)<br>0.12(f)	[1]
2	Ace2	GAGGAGAATGCCCAAAAGATGA	GAAATTTTGGGCGATCTTGGA	NM_001012006.2	hypothalamus	*0.03<br>*0.01(m)<br>*0.02(f)	[2]
3	At1A/Agtr1a	GAAATTTTGGGCGATCTTGGA	TAGATCCTGAGGCAGGGTGAAT	NM_030985.4	hypothalamus<br>placenta	0.06<br>0.2(m)<br>0.25(f)<br>0.24<br>0.44(m)<br>0.42(f)	[3]
4	At2-R/Agtr2	ACCTTTTGAACATGGTGCTTTG	GTTTCTCTGGGTCTGTTTGCTC	NM_012494.3	hypothalamus<br>placenta	0.24<br>0.29(m)<br>0.71(f)<br>0.2<br>0.18(m)<br>0.84(f)	[4]
5	Crh	CAGCCGTTGAATTTCTTG	GACTTCTGTTGAGGTTCC	NM_031019.2	placenta	0.15<br>0.32(m)<br>0.37(f)	[5]
6	Cxcl1	CCAAACCGAAGTCATAGCCA	CACCCTTTAGCATCTTTTGGA	NM_182952.2	hypothalamus<br>placenta	0.38<br>0.96	[6]
7	Dnmt1	CGGCTCAAAGACTTGGAAAG	TAGCCAGGTAGCCTTCCTCA	NM_053354.3	hypothalamus	0.42	[7]
8	Dnmt3a	TAGCCAGGTAGCCTTCCTCA	TGACGATGGAGAGGTCATTG	NM_001003957.1	hypothalamus	0.99	[7]
9	Dnmt3b	TAGGGTCCTGTCCCTGTTTG	GTGATTTTCCGGACGTCATT	NM_001396349.1	hypothalamus	0.62	[7]
10	Hdac1	GCGAGCAAGATGGCGCAGACT	GTGAGGCTTCATTGGGTGCCCT	NM_001025409.1	hypothalamus<br>placenta	0.35<br>0.32	[8]
11	Hdac3	ACCAGGCCTCCCAGCATGACA	CCGGGAAACACAGGGCAGTCG	NM_053448.2	hypothalamus<br>placenta	0.94<br>0.13	[8]
12	11ß-Hsd1	GAAGAAGCATGGAGGTCAAC	GCAATCAGAGGTTGGGTCAT	NM_017080.2	placenta	0.99	[9]
13	11ß-Hsd2	CGTCACTCAAGGGGACGTAT	TACAACGGGGCTAAGGTCAG	NM_017080.2	placenta	0.35	[9]
14a	Igf2	ATGTCACCCATGTCACCAAG	GGCTTGTGCCAATTAGGTTCT	NM_001190162.1	placenta		[10]
14b	Igf2	CGTGGCATCGTGGAAGAGT	ACGTCCCTCTCGGACTTGG	NM_001190162.1	placenta		[10]
15	IL1ß	GCAATGGTCGGGACATAGTT	AGACCTGACTTGGCAGAGGA	NM_031512.2	hypothalamus<br>placenta	0.39<br>0.18	[11]
16	IL6	TCTCTCCGCAAGAGACTTCCA	ATACTGGTCTGTTGTGGGTGG	NM_012589.2	hypothalamus<br>placenta	0.83<br>1.0(m)<br>0.65(f)<br>0.38	[12]
17	Lep	GAGACCTCCTCCATCTGCTG	CTCAGAGCCACCACCTCTGT	NM_013076.3	placenta	*0.05	[13]
18	Lepr	GCTGCTCGGAACACTGTTAAT	ACGGCATCCACTCTATATCCT	NM_012596.2	hypothalamus	0.24<br>0.31(m)<br>0.71(f)	[13]
19	Slc2a1	CTTCACTGTGGTGTCGCTGT	TTCAAAGAAGGCCACAAAGC	NM_138827.2	placenta	0.39<br>0.83(m)<br>0.66(f)	[14]
20	Slc2a3	GGTGGCTGGCTGTTGTAACT	AAGTCCTGCCTTTGGTCTCC	NM_017102.2	placenta	0.74<br>0.81(m)<br>*0.05(f)	[14]
21	TNFα	ACCACGCTCTTCTGTCTACTG	ACCACGCTCTTCTGTCTACTG	NM_012675.3	hypothalamus<br>placenta	0.66<br>0.23	[15]
22	Gpr43/Ffar2	TCGTGGAAGCTGCATCCA	GCGCGCACACGATCTTT	NM_001005877.1	hypothalamus		[16]
23	Gpr41/Ffar3	TGACGGTGAGCATAGAACGTTT	GCCGGGTTTTGTACCACAGT	NM_001108912.1	hypothalamus		[16]
24	Muc2	TGAGGTAGACAGAGCGACCA	GGAGTCCAAGCAGGGAGAG	XM_039101270.1	hypothalamus		[17]
25	Muc3	CTTGAGGAGGTGTGCAAGAAA	CCCCAGGGTGACATACTTTG	XM_039090116.1	hypothalamus		[18]
26	Muc4	GCTTGGACATTTGGTGATCC	GCCCGTTGAAGGTGTATTTG	XM_039088927.1	hypothalamus		[18]
27	Slc6a4/5Htt	TCTGAAAAGCCCCACTGGACT	TAGGACCGTGTCTTCATCAGGC	NM_013034.4	hypothalamus		[19]
28	Slc5a8/SMCT1	CTTCTGGGCTTGTTTTCTTTG	ATCGGGGGTCTAAGTTCTGTT	NM_001191987.1	hypothalamus		[20]

29	Slc5a12/SMCT2	ATTACCTTGACAGTGGCAGTG	CTTCCCAAAGAACATCCTGA	XM_032903870.1	hypothalamus	[20]
30	Glp-1r	CTTTGATGACTACGCCTGCT	CTTGGACTCTTCGCACTCC	NM_012728.2	hypothalamus	[21]

1. Gálvez-Prieto, B.; Bolbrinker, J., Stucchi, P.; de Las Heras, A.I.; Merino, B.; Arribas, S.; Ruiz-Gayo, M.; Huber, M.; Wehland, M.; Kreutz, R. and et al., Comparative expression analysis of the renin-angiotensin system components between white and brown perivascular adipose tissue. J. Endocrinol **2008**, 197, 55–64.
2. Kamilic, J.; Hamming, I.; Kreutz, R.; Bolbrinker, J.; Siems, W.E.; Nassar, I.; Sluimer, J.C.; Walther, T.; Navis, G.J. and van Goor, H. Renal ACE2 expression and activity is unaltered during established hypertension in adult SHRSP and TGR(mREN2)27. Hypertens Res **2010**, *33*, 123-8.
3. Aldubayan, M.A.; Ahmed, A.S.; Emara, A.M.; Ahmed, A.A. and Elgharabawy, R.M. Sinapic Acid Attenuates Cardiovascular Disorders in Rats by Modulating Reactive Oxygen Species and Angiotensin Receptor Expression. Oxid Med Cell Longev **2020**, *2020*, 1436858.
4. Lee, J.H.; Xia, S. and Ragolia, L. Upregulation of AT2 receptor and iNOS impairs angiotensin II-induced contraction without endothelium influence in young normotensive diabetic rats. Am J Physiol Regul Integr Comp Physiol **2008**, *295*, R144–54.
5. Briski, K.P.; Kale, A.Y. and Vavaiya, K.V. Impact of recurring intermediate insulin-induced hypoglycemia on hypothalamic paraventricular corticotropin-releasing hormone, oxytocin, vasopressin and glucokinase gene profiles: role of type II glucocorticoid receptors. Exp Brain Res **2009**, *195*, 499–507.
6. Saad, M.A.; Fahmy, M.I.; Sayed, R.H.; El-Yamany, M.F.; El-Naggar, R.; Hegazy, A.A. and Al-Shorbagy, M. Eprosartan: A closer insight into its neuroprotective activity in rats with focal cerebral ischemia-reperfusion injury. J. Biochem Mol Toxicol **2021**, *35*, e22796, https://doi.org/10.1002/jbt.22796.
7. Ishikawa, K.; Tsunekawa, S.; Ikeniwa, M.; Izumoto, T.; Iida, A.; Ogata, H.; Uenishi, E.; Seino, Y.; Ozaki, N.; Sugimura, Y., et al., Long-term pancreatic beta cell exposure to high levels of glucose but not palmitate induces DNA methylation within the insulin gene promoter and represses transcriptional activity. PLoS One **2015**, *10*, e0115350.8.
8. Dong, N.; B. Xu, and J. Xu, EGF-Mediated Overexpression of Myc Attenuates miR-26b by Recruiting HDAC3 to Induce Epithelial-Mesenchymal Transition of Lens Epithelial Cells. Biomed Res Int **2018**, *2018*, 7148023.
9. Zhou, H.Y.; Chen, X.X.; Lin, H.; Fei, A.L. and Ge, R.S. 11beta-hydroxysteroid dehydrogenase types 1 and 2 in postnatal development of rat testis: gene expression, localization and regulation by luteinizing hormone and androgens. Asian J Androl **2014**, *16*, 811–6.
10. Ye, X., Kohtz, A., Pollonini, G., Riccio, A. and Alberini, C.M. Insulin Like Growth Factor 2 Expression in the Rat Brain Both in Basal Condition and following Learning Predominantly Derives from the Maternal Allele. PLoS One **2015**, 10, e0141078.
11. Bae, G.D., Park, E.Y., Kim, K., Jang, S.E., Jun, H.S. and Oh, Y.S. Upregulation of caveolin-1 and its colocalization with cytokine receptors contributes to beta cell apoptosis. Sci Rep **2019**, *9*, 16785.
12. Hou, B., Zhao, Y., Qiang, G., Yang, X., Xu, C., Chen, X., Liu, C., Wang, X., Zhang, L. and Du, G. Puerarin Mitigates Diabetic Hepatic Steatosis and Fibrosis by Inhibiting TGF-beta Signaling Pathway Activation in Type 2 Diabetic Rats. Oxid Med Cell Longev **2018**, *2018*, 4545321.
13. Äijälä, M., Malo, E., Ukkola, O., Bloigu, R., Lehenkari, P., Autio-Harmainen, H., Santaniemi, M. and Kesäniemi, Y.A. Long-term fructose feeding changes the expression of leptin receptors and autophagy genes in the adipose tissue and liver of male rats: a possible link to elevated triglycerides. Genes Nutr **2013**, *8*, 623–35.
14. Merriman–Smith, R., Donaldson, P. and Kistler, J. Differential expression of facilitative glucose transporters GLUT1 and GLUT3 in the lens. Invest Ophthalmol Vis Sci **1999**, *40*, 3224–30.
15. Jiang, M., Li, C., Liu, Q., Wang, A. and Lei, M. Inhibiting Ceramide Synthesis Attenuates Hepatic Steatosis and Fibrosis in Rats With Non-alcoholic Fatty Liver Disease. Front Endocrinol (Lausanne) **2019**, *10*, 665.
16. Toral, M., Robles-Vera, I., De la Visitacion, N., Romero, M., Yang, T., Sánchez, M., Gómez-Guzmán, M., Jiménez, R., Raizada, M.K. and Duarte, J. Critical Role of the Interaction Gut Microbiota - Sympathetic Nervous System in the Regulation of Blood Pressure. Front Physiol **2019**, *10*, 231.
17. Han, K.S., Balan, P., Hong, H.D., Choi, W.I., Cho, C.W., Lee, Y.C., Moughan, P.J. and Singh, H. Korean ginseng modulates the ileal microbiota and mucin gene expression in the growing rat. Food Funct **2014**, *5*, 1506–12.
18. Turpin, W., Humblot, C., Noordine, M.L., Wrzosek, L., Tomas, J., Mayeur, C., Cherbuy, C., Guyot, J.P. and Thomas, M. Behavior of lactobacilli isolated from fermented slurry (ben-saalga) in gnotobiotic rats. PLoS One **2013**, *8*, e57711.
19. Erjavec, I., Bordukalo-Niksic, T., Brkljacic, J., Grcevic, D., Mokrovic, G., Kesic, M., Rogic, D., Zavadoski, W., Paralkar, V.M., Grgurevic, L., et al., Constitutively Elevated Blood Serotonin Is Associated with Bone Loss and Type 2 Diabetes in Rats. PLoS One **2016**, *11*, e0150102.

20. López-Barradas, A., González-Cid, T., Vázquez, N., Gavi-Maza, M., Reyes-Camacho, A., Velázquez-Villegas, L.A., Ramírez, V., Zandi-Nejad, K., Mount, D.B., Torres, N., et al., Insulin and SGK1 reduce the function of Na+/monocarboxylate transporter 1 (SMCT1/SLC5A8). Am J Physiol Cell Physiol **2016**, *311*, C720−C734.
21. Katsurada, K., Nandi, S.S., Zheng, H., Liu, X., Sharma, N.M. and Patel, K.P. GLP-1 mediated diuresis and natriuresis are blunted in heart failure and restored by selective afferent renal denervation. Cardiovasc Diabetol **2020**, *19*, 57.
